# Efficacy of ultra-short course chemotherapy for new smear positive drug susceptible pulmonary tuberculosis: study protocol of a multicenter randomized controlled clinical trial

**DOI:** 10.1186/s12879-017-2505-7

**Published:** 2017-06-19

**Authors:** Mengqiu Gao, Jingtao Gao, Jian Du, Yuhong Liu, Yao Zhang, Liping Ma, Fengling Mi, Liang Li, Shenjie Tang, on behalf of the Trial Team, Tang Shenjie, Li Liang, Gao Mengqiu, Liu Yuhong, Zhang Yao, Du Jian, Gao Jingtao, Ma Liping, Liu Rongmei, Ma Yan, Ding Xiaofen, Zhong Li, Xiao Hua, Shu Wei, Lv Zizheng, Guo Zhenyong, Wang Hong, Xie Shiheng

**Affiliations:** 1 Beijing Chest Hospital, Capital Medical University, Tuberculosis and Thoracic Tumor Research Institute, Beijing101149, Beijing, China; 2 Family Health International 360 (FHI360), Beijing, 100020 China

**Keywords:** Short course chemotherapy, Drug susceptible pulmonary tuberculosis, Clinical trial

## Abstract

**Background:**

Shortening the standard 6-month treatment for drug-susceptible pulmonary tuberculosis (DS-PTB) would be a major improvement for TB case management and disease control.

**Methods:**

We are conducting a randomized, open-label, controlled, non-inferiority trial involving patients with smear-positive, newly diagnosed DS-PTB cases nationwide to assess the efficacy and safety of two 4.5- month regimens in comparison to the standard 6-month WHO recommended regimen. The regimen used in one experiment group is a 4.5-month fluoroquinolone-containing regimen, which consists of full course of levofloxacin, isoniazid (H), rifampin (R), parazinamid (Z) and ethambutol (E). Regimen used in the second experiment group includes 4.5-month full course of H, R, Z, E with levofloxacin removed. Patients in the control group, receive H, R, Z and E for 2 months, followed by 4 months of H and R. The primary endpoint is treatment failure or relapse within 24 month after treatment completion.

**Discussion:**

Results from this trial along with other studies will contribute to the science of constructing a shorter, effective and safe regiment for TB patients.

**Trial registration:**

The protocol has been registered on ClinicalTrials.gov on 2 September,2016 with identifier NCT02901288.

## Background

Tuberculosis (TB) is now the leading infectious disease killer worldwide. In 2015, there were an estimated 10.4 million new TB cases worldwide. China is one of 30 TB high burden countries with third largest TB population globally. In 2015, 0.9 million new cases were estimated with an incidence of 67/100,000, accounting for 8.7% of the global total [1, 2]. Currently, 6-month short course remains the standard of care for the treatment of drug-susceptible pulmonary TB (DS-PTB), and its efficacy has been evaluated and established in controlled trials. Although standard 6-month regimens for drug- susceptible TB are highly efficacious, the duration of treatment is still considerably longer than that of most of respiratory infections. Poor adherence leads to treatment failure which in turn drives the emergence of drug resistance. Shortened treatment for DS-PTB would improve treatment outcomes and reduce costs. So regimens for TB that are shorter and/or simpler than the current 6-month regimen are needed.

The effectiveness of fluoroquinolones (FQ) in vitro, their sterilizing effect in both mice and human in vivo and their success in treating multi-drug resistant TB(MDR-TB) raised hope that if used as first line drugs [3–6], they might contribute to shortening therapy duration of DS-PTB. In 2014, 3 independent clinical trials named REMoxTB, RIFAQUIN and OFLOTUB/Gatifloxacin (Gfx) simultaneously reported final results of shortening treatment duration for DS-PTB by using FQ-based regimens [7–9]. Unfortunately, noninferiority for these regimens was not shown as compared to the standard 6-month regimen, indicating that shortening treatment to 4 months was not effective in these settings. Challenges as we faced, optimizing regimens for DS-PTB with shorter duration are still promising. The first pharmacodynamic data showed that levofloxacin(Lfx) was less effective than moxifloxacin(Mfx) and Gfx. Lfx was given at a dose less than 500 mg/day in these early studies [10]. With the prescription increase at the dose of 1000 mg/day in recent studies, Lfx showed the best early bactericidal activity as active as Mfx at 400 mg during the first 7 days of treatment [3]. Other studies have also shown that Mfx and Gfx are better than ofloxacin, but do not contain comparison with Lfx [11]. It seems to reveal that Lfx at high doses, rather than low doses, has better pharmacodynamic qualities than either Mfx or Gfx. Additionally, Lfx is less expensive, more widely available, and may be less likely to prolong the QT interval compared to later generation of FQs. Previous study showed that Z inhibited the ribosome-sparing process of trans-translation. Trans-translation is essential for freeing scarce ribosomes in nonreplicating organisms. Therefore, Z could eradicate persisting organisms which are the major cause of recurrence of TB [12]. If Z is given during the whole course, it can improve the sterilizing activities against *mycobacterial tuberculosis* (MTB) and reduce relapse of TB. On the basis of the supportive evidence, a multicenter randomized controlled clinical trial is currently in progress to evaluate whether 4.5 months of an Lfx-containing regimen will be as effective as 6 months of the standard regimen for the treatment of DS-PTB. Meanwhile, given the strong bactericidal and sterilizing activities of H, R, E and Z, parallel treatment shortening trial is also in progress with the consideration of increasing drug combination at continuation phase as compared with the standard 6-month regimen.

To our knowledge, preferable dosage and treatment duration of Lfx’s contribution combined with first line anti-TB drugs is not well studied. Neither is the shortening regimen with full length of treatment using H and R. This study aims to assess the efficacy and safety of 2 shortened regimens (study regimens) for DS-PTB compared to the WHO standardized 6-month regimen (control regimen). Success of the ultra-short course TB regimens would further shorten the treatment duration of DS-PTB, improve compliance and cure rates, reduce rates of adverse reactions and cost, as well as minimize the risk of drug resistance.

## Methods and Design

### Study design and oversight

We did a multicenter, randomized, controlled, open-label clinical trial within the framework of the China Tuberculosis Clinical Trial Consortium (CTCTC). The trial was sponsored by Ministry of Science and Technology of the People’s Republic of China and implemented by the leadership of Beijing Chest Hospital, Capital Medical University, together with other 34 TB specialized hospitals or Centers for TB Control and Prevention nationwide. The study protocol, the patient information sheets and consent forms were reviewed and approved by Central Ethics Committee of Beijing Chest Hospital, Capital Medical University/Beijing Tuberculosis and Thoracic Tumor Research Institute and the other participating medical facilities all approved the decision. The data and safety monitoring board planned to review efficacy and safety data at intervals of approximately 3 months throughout the study. Some of the trial medications were donated by Daiichi Sankyo Company Limited and Chengdu Jinhua Pharmaceutical Co.Ltd., but neither of these companies had any role in the study design, data accrual or further data analysis.

### Study patients

Adult patients who are newly diagnosed, previously untreated or treated less than 1 month with anti-TB drugs, are determined by chest X-ray with any abnormality consistent with TB and positive results of sputum smears on two occasions, with culture-confirmed susceptibility to H, R, E and Z and Lfx. All patients with written or witnessed oral informed consent were recruited if they met the trial eligibility criteria at screening. Detailed inclusion and exclusion criteria is provided in Table 1.

**Table 1:** Eligibility criteria

Inclusion criteria	Exclusion criteria
Is willing and able to give informed consent to participate in the trial treatment and follow-up (signed or witnessed consent if the patient is illiterate)	Is HIV positive
Is aged of 18–65 years old	Is concomitant with mental disorders
Is previously untreated or treated less than 1 month with anti-TB drugs	Is concomitant with diabetes while mellitus blood glucose out of control
Has 2 positive sputum smear results for mycobacterium TB at screening	Is pregnant or breastfeeding
Has a chest X-ray abnormality compatible with a diagnosis of PTB	Has a prolonged QTcF^a^ (>480 ms)
Is not pregnant for female patient at bearing age and willing to use effective contraception during study period	Is currently participate in another trial of a medicinal product
Has CrCl^b^ > 30 ml/ min, HB^c^ > 7.0 g/dL and PLT^d^ > 50 × 10^9^/L at screening	Is concomitant with severe cardiovascular, hepatic, renal, nervous system, hematopoietic system injury and other diseases, or concomitant with neoplastic diseases
Has ALT^e^ and TBIL^f^ less than 2 times the upper limit of normal at screening	Has extensive lesion in the lung concomitant with respiratory insufficiency
	Has known allergy to any of the anti-TB drugs in the trial
	Is taking any medications contraindicated with the medicines in any trial regimen of the study

^a^ *QTcF* Fridericia-corrected QT, ^b^ *CrCl* creatinine clearance, ^c^ *HB* hemoglobin, ^*d*^ *PLT* Platelet, ^e^ *ALT* alanine aminotransferase, ^f^ *TBIL* total bilirubin

### Randomization and study regimens

Eligible patients who have provided written or witnessed oral informed consent were randomly assigned a unique study number sequentially on a web-based computerized algorithm, with the use of blocks of variable sizes which were stratified according to the study sites. Patients were randomized in a ratio of 1:1:1 to one of three regimens: a control regimen 2HREZ/4HR, which consists of 6 months of H and R administered daily, supplemented by E and Z in the first 2 months; a 4.5-month Lfx-containing regimen 4.5LfxHREZ, which is composed of H, E, Lfx, R, and Z administered daily, throughout the length of therapy; and another 4.5-month regimen4.5HREZ with Lfx removal from the former regimen. In all three groups, drug doses were adjusted according to patient’ weight, as described in Table 2. Treatment is directly observed at each local health facility and supervised by patient-friendly App management tools.

**Table 2:** Anti-TB drugs and their dosage used in the trial

Drugs (Abbreviation)	Formulation	Dosage Daily	
		<50 kg	≥ 50 kg
Isoniazid (H)	100 mg/tablet	300 mg	300 mg
Rifampicin (R)	150 mg/ tablet	450 mg	600 mg
Ethambutol (E)	250 mg/ tablet	750 mg	1000 mg
Pyrazinamid (Z)	250 mg/ tablet	1500 mg	30 mg/kg
Levofloxacin (Lfx)	100 mg/ tablet	600 mg	800 mg

### Sample size assumption

Under the conditions of the trial, we assumed that the standard 6-month regimen has a 95% or higher sputum culture conversion rate and with estimated 92.5% of that for 4.5-month study regimen based on the published data. The study was designed to determine whether either of the shortened study regimens was not inferior to the control regimen and a margin of noninferiority of 5% was used. With the assumption that as many as 20% of patients will loss to follow-up or not be assessable in the primary analysis, and with the consideration of approximately 5.5% primary drug resistance prevalence, a total target sample size was 3900 patients (1300 subjects per group) would be required to demonstrate noninferiority, with a statistical power of 0.8 and an alpha level of 0.05 (one-sided). Participant recruitment began in August 2016 and is expected to finish in December 2018.

### Data management

All procedures conform to confidentiality standards for medical data. Authorized medical staff treating the patient is granted unlimited access to the patients’ data, whereas restricted access to anonymized data is granted to other staff and researchers. All data will be entered electronically into specific database developed by CTCTC for this study within 1 week and all original forms or records will be kept at all study sites. Participant trial folders are stored in order and in a secure and accessible place at each study site which will be maintained in storage for a period of 3 years after completion of the study. All data will be performed periodically on the CTCTC backup server.

### Statistical analysis

According to the standard approach of noninferiority trials, the analysis will be conducted with both per-protocol (PP) and modified intention-to-treat (mITT) populations. Noninferiority, defined as a between-group difference of less than 5% in the upper boundary of one-sided 90% Wald confidence interval (which is equivalent to a one-sided significance of 5%) for the proportion of patients with primary unfavorable outcomes, must be shown on both analyses above to declare that either regimen is noninferior. We will use the chi-square test to compare the secondary outcomes of patients’ sputum-culture conversion at the end of 8 weeks (intensive phase) and treatment completion across treatment groups and the log-rank test to compare the time to culture-negative status. Other secondary outcomes will be analyzed with similar methods, including the time to an unfavorable outcome, the outcome at the end of treatment, adverse effect occurring frequency, patients’ adherence and the transition of radiological manifestation. Missing data will be dealt with the last observation carried forward imputation technique. Dropouts and withdrawals from the study will be recorded through the intervention and follow-up periods. When differences in baseline phenotype are present, these differences will be taken into account during analysis of treatment effect between groups, by regarding them as covariates.

### Outcome measures

The primary efficacy outcome measures include the percentage of participants with treatment failure or relapse by 24 months after the end of treatment, percentage of participants with treatment failure at either 4.5 months or 6 months after randomization. The secondary efficacy and safety outcomes include the time to sputum (both smear and culture) conversion within intensive phase of 2 months and the percentage of participants with sputum conversion (both smear and culture) at the completion of treatment, the number of adverse reactions occurring on treatment and during the follow-up period, adherence to treatment and radiological manifestation transition. Of note, treatment failure was defined as sputum smear positive at the end of treatment and relapse after completion of treatment is defined as at least one positive culture in the scheduled follow-up visits in two years. Participants with negative cultures at the end of follow-up are considered to have had a favorable outcome. For all treatment failures and relapses, one culture of a sputum sample obtained before treatment and one culture of a sputum sample obtained after treatment failure or relapse are planned to store at −80 °C and the samples will be sent to national clinical TB reference lab in Beijing Chest Hospital for further research. The detailed observation schedule is present in Table 3.

**Table 3:** Assessment schedule – for all patients recruited

Observation/Investigation		Screening	Treatment Phase (weeks)									Follow-up (Post-treatment phase, months)							
Study visits		0	2	4	6	8	12	16	19	22	26	3	6	9	12	15	18	21	24
Time window (days)			±3	±3	±3	±3	±7	±7	±7	±7	±7	±14	±14	±14	±14	±14	±14	±14	±14
Demographics		X																	
Written or witnessed informed consent		X																	
Patients eligible criteria judgement		X																	
Medical history and clinical assessment		X	X	X	X	X	X	X	X	X	X	X	X	X	X	X	X	X	X
Concomitant diseases and medication taken assessment		X	X	X	X	X	X	X	X	X	X	X	X	X	X	X	X	X	X
Randomized assignment		X																	
Efficacy evaluation																			
Bacteriological test	Sputum smear	X	X	X	X	X	X	X	X	X	X	X	X	X	X	X	X	X	X
	Sputum culture	X	X	X	X	X	X	X	X	X	X	X	X	X	X	X	X	X	X
X-ray		X				X			X		X		X		X		X		X
Safety evaluation																			
Complete blood count		X		X		X	X	X	X	X	X								
Urinalysis		X		X		X	X	X	X	X	X								
Hepatic and renal functions		X		X		X	X	X	X	X	X								
Electrocardiograph (ECG)		X		X		X	X	X	X	X	X								
Body weight		X	X	X	X	X	X	X	X	X	X	X	X	X	X	X	X	X	X
Adverse effect record			X	X	X	X	X	X	X	X	X	X	X	X	X	X	X	X	X
Others																			
Drug distribution log		X	X	X	X	X	X	X	X	X									
Drug retrieve log			X	X	X	X	X	X	X	X	X								

X indicates assessments required at particular visits

### Safety Reporting

Adverse events are defined and reported according to the Common Terminology Criteria for Adverse Events 4.03 (CTCAE). An adverse event is categorized as serious if it led to death, permanent or significant disability, a congenital anomaly or birth defect, life threatening, or required hospital admission for management. A data monitoring committee is needed with the consideration of the potential risk of this study at regular intervals during the trial. Interim analysis is planned when one third of enrolled patients have finished treatment. Thus efficacy and safety data of the study will be reviewed. The study can be stopped before reaching sample size if the data monitoring committee recommends termination of the study or termination of one of the treatment regimens due to unacceptable levels of treatment failure, relapse, adverse effect or mortality compared to the control arm. Important protocol modifications during this study will be updated to the trial registry online.

### Clinical site monitoring and quality assurance

Family Health International 360 China office (FHI360) is responsible for monitoring data quality in accordance with trial Standard Operating Procedures (SOPs). Based on the monitoring plan, field visit and audit will be performed at different stages. All participant records, CRFs, and other source documents for the patients recruited in this study will be made available for review by the monitors. The investigator and staff are responsible for being present and available for consultation during scheduled site audit visits. A site field visit feedback will be sent to each study site made by authorized individuals. Investigators of each local site will be convened monthly via web-based remote conference system to share with the progress of study and discuss with the problems met during the trial searching for trouble shooting answers. Quality assurance (QA) procedures are carried out according to Quality Management documents. A review of these documents is undertaken by Quality Management Advisory Group (QMAG) of CTCTC. QMAG has the power to stop ongoing practices of any site which violates study protocol significantly and makes decision of its restarting or closing with respect to specific circumstance. Quality control (QC) procedures are performed at each study site by designated staff to guarantee all the practices comply with trial SOPs. Microbiology examinations are also under proper QC guideline developed by CTCTC. Moreover, Good Clinical Practice (GCP) training and appropriate Good Laboratory Practice (GLP) training will be provided for all staff involved in the trial; this will be a component of capacity strengthening of the trial.

## Discussion

Current 6-month regimens for new smear positive DS-PTB are used in more than 90 countries and are designed and evaluated cost-effective [13]. With the exciting results of shortened regimens for MDR-TB cases from the previous 24 months to 9 months length of therapy [14–16], researchers have been paving the way to explore the probably shorter regimens for DS-PTB inferior to the current standard ones. In the 1970s, the introduction of R resulted in a 15 to 20% increase of sputum conversion at 8 weeks and shortening duration of treatment from 18 months to 9 months. The later introduction of Z led to a further 13% increase of sputum conversion at 8 weeks, allowing therapy to be further reduced from 9 months to 6 months [17–20]. Based on these findings, Medical Research Council (MRC) led the controlled clinical trial of five short-course (4-month) chemotherapy regimens for PTB patients including 2HRSZ/2HRZ, 2HRSZ/2HR, 2HRSZ/2HZ, 2HRSZ/2H, 2HRZ/2H. However, all the shortened regimens failed to present expected primary outcome, instead, with 16%, 11%, 32%, 30% and 40% recurrence after 24 months of treatment completion [21, 22]. For years with new drugs progress for TB chemotherapy, especially the favorable pharmacodynamics and bactericidal activity of FQ on MTB, shortening the treatment duration for DS-PTB by using a FQ-based regimen is of great hot topic 3 independent shortening clinical trials have been reported simultaneously with a failure to show noninferiority to the 6-month standard regimen attributed to the higher recurrence rate observed with the 4-month regimens, despite rapid culture conversion during treatment [7–9]. These findings raised questions that the shortening FQ-containing regimens did not work as adequately as the murine model did which may overpredict the sterilizing potency of the regimens.

Since Lfx administered with high dose is of comparable efficacy compared with Mfx and Gfx against MTB, it is possible to introduce Lfx to the regimen for shorter duration of DS-PTB. However, there is also concern that Lfx may fail as first-line anti-TB drugs because of its widely applied for common infections such as urinary and gastrointestinal tracts, wounds, and other lung infections. In high-TB-burden countries, a great number of cases with undiagnosed TB are likely to take an FQ, which could be selected for resistance in at least a fraction of MTB they harbor [23]. Several studies focus on FQ resistance and its association with previous FQ exposure indicated a low prevalence. Researchers from Korea found 2.6% FQ resistance in patients exposed to FQs while 3.4% resistance in patients without FQ exposure [24]. A study from Taiwan found no correlation of FQ resistance with either FQ exposure or duration of FQ exposure, but saw a positive correlation with previous anti-TB treatment and resistance to any other drugs [25]. Previous study from the same research team comparing sputum taken both before and after a course of FQs treatment showed that patients who received an FQ before standard anti-TB treatment had a poor prognosis, most likely as a result of the emergence of drug resistant. 11.1% of the MTB obtained FQ resistance after taking an FQ for 1–3 weeks [26]. The fifth national TB epidemiology survey in China of 2010 reported that Lfx resistant TB accounted for 9.1% among new TB patients [27]. A high prevalence of FQ-resistant TB may void the possibility of Lfx as first line drug for shortening DS-PTB treatment duration. Appropriate bacteriological and histopathological tests for TB should be performed as early as possible before Lfx-containing regimen is recommended. If the study regimen is successful, it is expected to provide a new standard of care for DS-PTB which would cut down the number of required clinic visits and the burden on the health care system and could also decrease the percentage of patients who fail to complete the full course of current longer therapy. If the study regimens are shown to be noninferior or superior to the control regimen, that would represent an even greater advance for patients with DS-PTB and TB control programmes globally. However, there are a few negative aspects that need to be considered before an FQ-containing first-line regimen could be broadly recommended. The FQs are fairly effective drugs for common nonspecific respiratory syndromes and community-acquired pneumonia, and curtailing this usage to ensure they remain effective as first-line TB drugs may not prove beneficial to all-cause morbidity and mortality at the community level. Also, using FQs as standard first-line therapy would reduce their effectiveness against MDR-TB, and could perhaps result in the emergence of more XDR-TB. Its implementation might be suggested only where the prevalence of Lfx-resistant TB is low and FQs are not routinely used for nonspecific respiratory symptoms when TB cannot be effectively excluded would reduce concerns above.

## Abbreviations

CTCTC: China Tuberculosis Clinical Trial Consortium
DS-PTB: Drug-susceptible pulmonary tuberculosis
E: Ethambutol
FHI360: Family Health International 360
FQ: Fluoroquinolones
GCP: Good Clinical Practice
Gfx: Gatifloxacin
GLP: Good Laboratory Practice
H: Isoniazid
Lfx: Levofloxacin
MDR-TB: Multi-drug resistant TB
Mfx: Moxifloxacin
MRC: Medical Research Council
MTB: Mycobacterial tuberculosis
QA: Quality assurance
QMAG: Quality Management Advisory Group
R: Rifampin
TB: Tuberculosis
Z: Parazinamid
